# Low Temperature CO oxidation over Iron Oxide Nanoparticles Decorating Internal Structures of a Mesoporous Alumina

**DOI:** 10.1038/srep40497

**Published:** 2017-01-16

**Authors:** Il Hee Kim, Hyun Ook Seo, Eun Ji Park, Sang Wook Han, Young Dok Kim

**Affiliations:** 1 Department of Chemistry, Sungkyunkwan University, Suwon, 440-746, Republic of Korea

## Abstract

Using a chemical vapor deposition method with regulated sample temperatures under ambient pressure conditions, we were able to fully decorate the internal structure of a mesoporous Al_2_O_3_ bead (~1 mm in particle diameter) with iron oxide nanoparticles (with a mean lateral size of less than 1 nm). The iron oxide-decorated Al_2_O_3_ showed a high CO oxidation reactivity, even at room temperature. Very little deactivation of the CO oxidation activity was observed with increasing reaction time at ~100 °C. Additionally, this catalyst showed high CO oxidation activity, even after annealing at ~900 °C under atmospheric conditions (i.e., the structure of the catalysts could be maintained under very harsh treatment conditions). We show that our catalysts have potential for application as oxidation catalysts in industrial processes due to the simplicity of their fabrication process as well as the high and stable catalytic performance.

Heterogeneous catalysis for the oxidation of various chemical compounds is one of the core technologies in advanced environmental science and engineering. Harmful compounds, such as volatile organic compounds and carbon monoxide, can be generated from vehicles and industrial sources due to the incomplete combustion of fossil fuels. These compounds should either be captured^1^,^2^,^3^ or converted into non-toxic CO_2_ and H_2_O before they are emitted into the air^4^,^5^,^6^. Heterogeneous catalysis is advantageous over the use of adsorbents because adsorbents must be frequently replaced or regenerated, whereas heterogeneous catalysts can, in principle, be used semi-permanently.

CO oxidation has been widely studied in the fields of surface science and heterogeneous catalysis due to its technological importance and fundamental interest^7^,^8^,^9^,^10^,^11^,^12^,^13^,^14^,^15^,^16^,^17^,^18^. It has been widely reported that Pt-group metals, such as Pt, Pd, Rh, and Ir, can show high activity for catalytic CO oxidation^5^,^7^,^12^,^13^,^14^,^15^,^18^,^19^. In fact, most of the commercially-available exhaust catalysts rely on Pt-group metals^20^,^21^.

One of the challenging issues in developing CO oxidation catalysts is decreasing the operation temperature of the catalysts. Typically, Pt-group metals only show sufficiently high activity for CO oxidation above ~150 °C, which means that the catalytic oxidation of CO does not fully work at lower temperatures (e.g., room temperature)^13^,^22^. Therefore, pollutants in the exhaust gas can be emitted and contaminate the atmosphere before the catalytic converter reaches the operating temperature of the catalysts^22^. Another important issue is the high cost of Pt-group metals and their low natural abundance, which may limit future applications of these metals^23^.

As alternatives to Pt-group metals for CO oxidation, Au nanocatalysts have been considered^22^,^24^,^25^,^26^. Au nanoparticles (less than 5 nm) were shown to be reactive for CO oxidation, even below room temperature, and have drawn interest over the last three decades^22^,^24^,^25^,^26^. Particularly, supported and unsupported Au nanoparticles and clusters are interesting from a fundamental point of view due to their pronounced particle size effect on the catalytic activity: a drastic increase in the turn-over-frequency is observed as the particle size is reduced below 3–4 nm^22^,^24^,^25^,^26^. A drawback of Au-based nanocatalysts is their low stability. At higher temperatures, Au nanoparticles can agglomerate to form larger entities, which show drastically decreased catalytic activity^27^,^28^,^29^. Oxidation catalysts should be able to operate at lower temperatures and sustain their structure and catalytic activity at higher temperatures (~900 °C)^20^. In addition to this stability issue, Au is not a particularly economic material^30^.

More recently, more cost-effective materials with higher stability have been considered as CO oxidation catalysts. Catalysts based on nanostructures of Cu^31^,^32^, Ni^33^,^34^,^35^, and Co^36^,^37^ compounds were shown to catalyze CO oxidation at room temperature. Again, the development of more stable catalysts continues to be a challenging issue; small nanoparticles (less than ~10 nm in size), which are catalytically active due to their high surface areas and abundance of catalytically-active, under-coordinated sites (e.g., edges), are not stable and tend to easily form larger particles^34^,^35^.

In order to increase the stability of nanocatalysts, attempts have been made to incorporate catalytically-active nanostructures into mesoporous supports. Catalytically-active nanostructures were either introduced into the mesoporous structure during the synthesis of mesoporous structures^32^,^38^,^39^,^40^,^41^ or nanoparticles were post-incorporated into the pre-formed mesoporous template^34^,^35^,^42^,^43^,^44^,^45^. The latter method is advantageous in that commercially-available and cost-effective mesoporous materials can be utilized; recently, atomic layer deposition (ALD) has been used to incorporate catalytically-active nanoparticles into mesoporous structures^34^,^35^,^42^,^43^,^44^. For example, Pt nanoparticles were incorporated into carbon aerogels (resulting in high activity for CO oxidation)^42^ and NiO nanoparticles were deposited in mesoporous SiO_2_ and Al_2_O_3_ (resulting in highly stable catalytic activity for various heterogeneously-catalyzed reactions)^34^,^35^. It has often been observed that ALD successfully deposits nanoparticles on both the outermost surface and also into the deeper parts of the mesoporous material with a diffusion depth of several micrometers^34^,^35^,^42^,^43^. An alternative to ALD is the vapor infiltration method, in which mesoporous templates are exposed to the vapor of inorganic precursors under vacuum conditions for extended periods of time and then subsequently exposed to other oxidizing or reducing agents^45^,^46^. Unlike ALD, such infiltration technologies do not allow precise control over the amount of material that is deposited; however, the preparation of nanoparticles within mesoporous substrates is much simpler due to the absence of alternating and time-consuming exposing/purging steps of dissimilar precursors (as is done in ALD).

In the present work, we show a one-pot strategy for the incorporation of catalytically-active metal oxide nanoparticles into the pre-formed mesoporous substrate, referred as temperature-regulated chemical vapor deposition (TR-CVD)^47^. Iron oxide nanoparticles, formed by the evaporation and deposition of a ferrocene precursor under ambient conditions without a vacuum system, can fully decorate the internal structure of a mesoporous Al_2_O_3_ bead with a mean pore diameter of ~12 nm and bead diameter of 1 mm. Here, the oxygen and water vapor originally present in natural air act as the oxidizing agents of ferrocene to form iron oxide. High CO oxidation reactivity and structural stability of the iron oxide-decorated Al_2_O_3_ bead are demonstrated.

## Results and Discussion

### Characterization of Fe_2_O_3_/Al_2_O_3_

**Figure 1:**
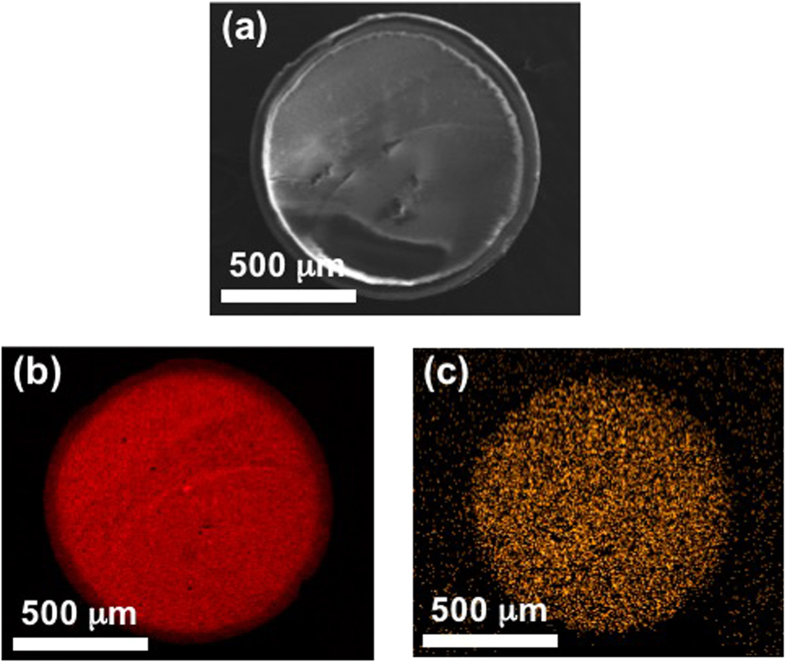
(**a**) SEM image of Fe_2_O_3_/Al_2_O_3_, and EDS mapping image of (**b**) Al and (**c**) Fe of Fe_2_O_3_/Al_2_O_3_ sample prepared by TR-CVD. Fe_2_O_3_/Al_2_O_3_ sample with size of ~1 mm was mechanically fractured, and the internal structure of the sample was studied (**a**). Al species is evenly distributed over the entire cross-sectional plane (**b**), and Fe species is also evenly distributed over the entire cross-sectional plane.

We deposited iron oxide on a spherical Al_2_O_3_ beads with a diameter of ~1 mm using TR-CVD process and they were mechanically fractured into two hemispheres and cross-sectional scanning electron microscopy (SEM) (Fig. 1a) image was obtained together with energy dispersive spectroscopy (EDS) mapping images (Al in Fig. 1b and Fe in Fig. 1c). The red trace in Fig. 1b represents the Al species, which is the main element of the Al_2_O_3_ support, and it is evenly distributed over the entire cross-sectional plane of Fe_2_O_3_/Al_2_O_3_. The Fe species represented by the orange color in Fig. 1c is also evenly distributed over the entire cross-sectional plane, indicating that Fe was deposited on inner parts of the 1 mm-sized Al_2_O_3_ substrate as well as its outer surface.

The surface area and average pore diameter of the bare Al_2_O_3_, and Fe_2_O_3_/Al_2_O_3_ samples annealed at 450, 600, 750, and 900 °C were obtained through Barrett-Joyner-Halenda (BJH) and Brunauer-Emmett-Teller (BET) method after the N_2_ adsorption/desorption isotherms (Supplementary Fig. 1 and Supplementary Table 1). The surface area and average pore diameter of the samples did not show significant changes after iron oxide deposition and subsequent annealing. It indicates that the mesoporous structure of Al_2_O_3_ was maintained after Fe_2_O_3_ deposition even after the annealing process at 900 °C. It is worth mentioning that the Fe content of these samples was estimated to be 5.39 wt% by inductively coupled plasma optical emission spectrometry (ICP-OES) analysis, i.e., a relatively large amount of Fe homogeneously decorated the internal surface of the entire mesoporous structure of Al_2_O_3_ bead via a TR-CVD process and it did not clog the mesopores of Al_2_O_3_ even after the annealing process at 900 °C.

**Figure 2:**
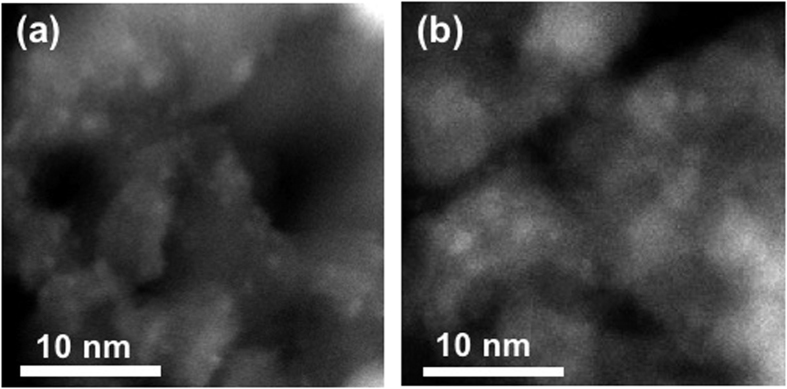
TEM-HAADF image of (**a**) 450 °C, and (**b**) 750 °C annealed Fe_2_O_3_/Al_2_O_3_. In Fe_2_O_3_/Al_2_O_3_ sample, Fe_2_O_3_ nanoparticles are uniformly distributed (**a**,**b**), and as annealing temperature increased from (**a**) 450 °C to (**b**) 750 °C, the particle size of Fe_2_O_3_ increased.

In order to elucidate the structure of the Fe species deposited on Al_2_O_3_, the Fe-deposited sample was ground, annealed at 450 and 750 °C, and analyzed by high-angle annular dark field (HAADF) scanning transmission electron microscopy (STEM) images (Fig. 2). Small nanoparticles with a diameter of about 1 nm can be distinguished in TEM images as small white dots and the average size of nanoparticles increased from 0.81 nm to 0.98 nm as the annealing temperature increased from 450 to 750 °C (Supplementary Fig. 2). EDS analysis confirmed that small white dots were iron-related species (Supplementary Fig. 3) and the results of XPS analysis which will be shown in later parts of manuscript revealed that most of iron-related species were in Fe (III) states. However, no lattice fringes corresponding to Fe_2_O_3_ were observed at white dots in TEM images and no iron-related peaks were found in X-ray diffraction (XRD) patterns of Fe-deposited Al_2_O_3_ annealed at 450 and 750 °C. These can be attributed to the small size of nanoparticles of Fe_2_O_3_ as well as their low crystallinity (Supplementary Fig. 4). It is worth mentioning that NiO nanoparticles deposited on mesoporous SiO_2_ via atomic layer deposition initially had a size less than 2 nm in average diameter, but it agglomerated into larger particles with a diameter of ~20–30 nm upon the annealing process at 750 °C^43^. In contrast to the case of the NiO on porous SiO_2_, Fe_2_O_3_ on mesoporous Al_2_O_3_ sustained its particle diameter (of less than ~1 nm) upon the annealing at 750 °C (i.e., nanoparticles of Fe_2_O_3_ on Al_2_O_3_ showed a higher thermal stability).

**Figure 3:**
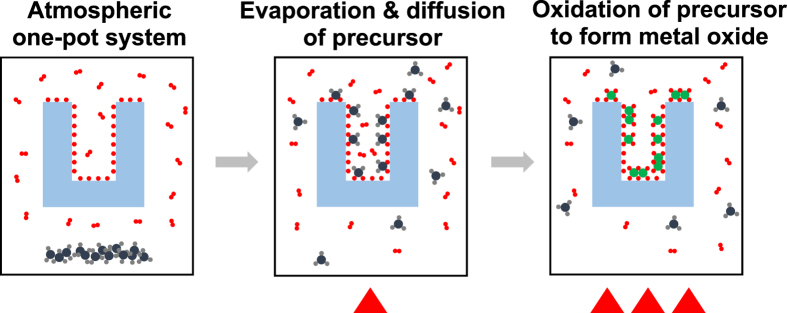
A schematic diagram of TR-CVD process.

These data reveal that iron oxide was deposited on the surface of the mesoporous Al_2_O_3_ bead via TR-CVD, even at the core region of the Al_2_O_3_ substrate (with an average diameter of 1 mm). Additionally, the mesoporous structure and high surface area of Al_2_O_3_ were maintained during the iron oxide deposition. The TR-CVD is regarded as a sequential process of the following two steps:

- Vaporization of Fe(Cp)_2_, its diffusion into the deeper part of the mesoporous Al_2_O_3_, and its adsorption onto the surface of Al_2_O_3_ at lower temperatures.
- Oxidation of the adsorbed metal precursor into the metal oxide and oxidative removal of the organic ligands of Fe(Cp)_2_ at higher temperatures (i.e., 200 °C) (Fig. 3).

A temperature of <100 °C was high enough for Fe(Cp)_2_ to be evaporated but not high enough for Fe(Cp)_2_ to react with oxygen and water vapor in the atmosphere; this allowed further diffusion of the Fe(Cp)_2_ vapor into the core of the mesoporous Al_2_O_3_ bead without clogging the pores. When the temperature of the reactor was increased to 200 °C, however, Fe(Cp)_2_ reacted with the oxygen and water vapor inside of the reactor to form iron oxides on the surface of Al_2_O_3_. Fe(Cp)_2_ precursors might not be fully oxidized forming Fe_2_O_3_ (Fe III) at 200 °C, considering that the typical ALD temperature window for Fe_2_O_3_ deposition using Fe(Cp)_2_ and oxygen precursors is 350~500 °C^48^,^49^. However, iron oxides deposition by TR-CVD was followed by annealing processes at higher temperatures (>450 °C) which were high enough to fully oxidize iron oxides producing the Fe_2_O_3_ on Al_2_O_3_ substrate. Our XPS analysis results revealed that most of Fe ions of catalyst were in fully oxidized states (Fe III) after the annealing processes (>450 °C). It is worth to emphasize that our strategy (TR-CVD) for the incorporation of a metal oxide into a mesoporous substrate is much simpler and more effective than ALD, which has been widely used for incorporating metal and metal oxide nanoparticles into mesoporous substrates. Note that ALD requires a high vacuum system and repeated on-off control of the inlet valves of different precursors; this process is much more complicated than the TR-CVD method presented here. Unlike many conventional infiltration techniques, we do not use a high vacuum system, which can be advantageous for larger-scale production. However, our proposed method can only be used when the inorganic precursor is sufficiently inert to hinder homogeneous reactions between the vapors of the inorganic precursor and air present in the gas phase. Here, we show that our TR-CVD process works under ambient conditions to form iron oxide from Fe(Cp)_2_. A broader search of other possible inorganic precursors for application with ambient pressure TR-CVD will be conducted in the future.

### Catalytic activity of Fe_2_O_3_/Al_2_O_3_

**Figure 4:**
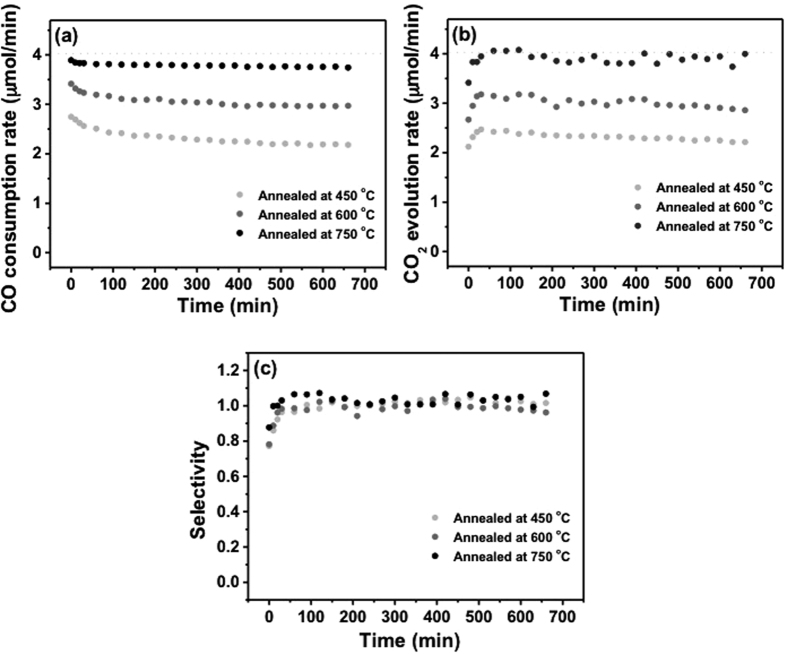
(**a**) CO consumption rate, (**b**) CO_2_ evolution rate, and (**c**) CO_2_ selectivity of Fe_2_O_3_/Al_2_O_3_ annealed at 450, 600, and 750 °C. Dashed lines indicate the respective values corresponding to 100% conversion of CO to CO_2_.

After annealing of the TR-CVD prepared Fe_2_O_3_/Al_2_O_3_ samples at 450, 600, and 750 °C for 2 h, the catalytic activity of Fe_2_O_3_/Al_2_O_3_ for CO oxidation was tested at a reaction temperature of 100 °C for 2 h (Fig. 4). The CO consumption and CO_2_ evolution rates of each sample were plotted as a function of the reaction time in Fig. 4a and b, respectively. As the annealing temperature increased from 450 to 750 °C, the catalytic activity of Fe_2_O_3_/Al_2_O_3_ gradually increased and the deactivation of catalytic activity over the reaction time became less pronounced. For all of the Fe_2_O_3_/Al_2_O_3_ catalysts, the CO_2_ selectivity was 1 for 12 h except for the initial 50 min (Fig. 4c) indicating that most of the CO was oxidized to CO_2_ by the catalysts.

From the literature, NiO nanocatalysts prepared by ALD and supported by mesoporous Al_2_O_3_ and SiO_2_ showed the highest catalytic activity after annealing at 450 °C, and the activity decreased as the annealing temperature increased from 450 to 750 °C^34^,^35^. In contrast, the activity of the Fe_2_O_3_/Al_2_O_3_ prepared by TR-CVD increased as the annealing temperature increased from 450 to 750 °C.

**Figure 5:**
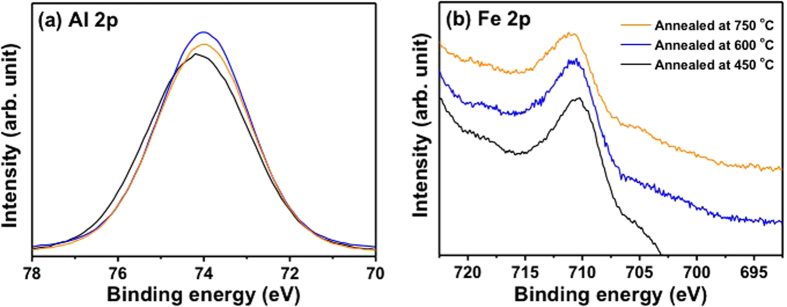
(**a**) Al 2p and (**b**) Fe 2p spectra of Fe_2_O_3_/Al_2_O_3_ annealed at 750 °C. The binding energies of XPS spectra of three samples were calibrated with the respective C 1 s peak (284.5 eV), and the intensities of Al 2p and Fe 2p peaks were normalized by respective Al 2p peak area.

In order to determine the origin of the increased catalytic activity of Fe_2_O_3_/Al_2_O_3_ as the annealing temperature increased (from 450 to 750 °C), X-ray photoelectron spectroscopy (XPS) analyses of Fe_2_O_3_/Al_2_O_3_ were conducted after annealing at 450, 600, and 750 °C (Fig. 5). The binding energies of XPS spectra of three samples were calibrated with the respective C 1 s peak (284.5 eV). The intensities of Al 2p and Fe 2p core-level XPS peaks were normalized by respective Al 2p peak area. The Al 2p peak of the Fe_2_O_3_/Al_2_O_3_ annealed at 450 °C was centered at 74.2 eV and it became sharper with a slight negative shift (from 74.2 eV to 73.9 eV) as the annealing temperature of the catalysts increased from 450 to 750 °C (Fig. 5a). Whereas the Fe 2p peaks shifted to a higher binding energy region with increasing annealing temperature (Fig. 5b).

**Figure 6:**
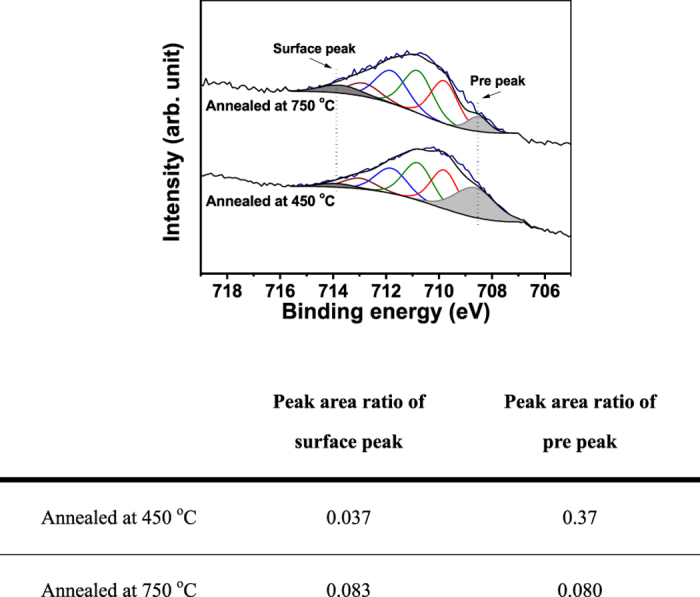
Fe 2p_3/2_ core-level XPS spectra of Fe_2_O_3_/Al_2_O_3_ catalyst annealed at 450 and 750 °C, and the table for the ratio values of surface peak and pre peak with respect to bulk Fe (III) state.

In order to elucidate the chemical environments of Fe species in samples with different annealing treatments, the Fe 2p spectra of the samples annealed at 450 and 750 °C was convoluted using six different components of the linearly-combined Gaussian-Lorentzian function (Fig. 6). It is well known that the Fe 2p core level spectrum of Fe(III) species shows complicated features due to the multiplet splitting that is based on various physical origins^50^,^51^. The lowest binding energy peak is often referred to as a pre-peak and corresponds to Fe ions with a lower oxidation state, as compared to the normal oxidation state. The four next highest binding energy peaks are from multiplet splitting of the Fe(III) species, as suggested by Gupa and Sen^51^. They predicted these peaks based on the electrostatic interaction and spin-orbit coupling between the 2p core hole/unpaired 3d electrons of the photoionized Fe ion and crystal field interaction^50^. The highest binding energy peak, which is centered at ~714 eV, can be assigned to the surface Fe(III) species of bulk Fe_2_O_3_. The area ratio of surface Fe(III) peak and pre-peak in a lower oxidation state with respect to those of the respective bulk Fe(III) states of 450 and 750 °C-annealed catalysts are summarized in Fig. 6. It can be clearly seen that annealing at 750 °C significantly increased the peak intensity of the surface Fe(III) of bulk Fe_2_O_3_ and simultaneously reduced the intensity of pre-peak in lower oxidation states compared to the case of 450 °C annealing. Based on our experimental results, the higher catalytic activity of 750 °C-annealed Fe_2_O_3_/Al_2_O_3_ than that of 450 °C-annealed sample can be attributed to a higher number of surface Fe(III) states and a lower portion of Fe ions in a lower oxidation state in iron-oxide particles of 750 °C-annealed sample compared to the case of 450 °C-annealing. We would like to mention that 750 °C-annealed Fe_2_O_3_/Al_2_O_3_ catalyst showed higher uptake of H_2_ than 450 °C-annealed sample in the temperature range of 100~450 °C during the H_2_-Temperature programmed reduction (TPR) experiments (Supplementary Fig. 5). Particularly the amount of H_2_ uptake of 750 °C-annealed sample was more than 4 times higher than that of 450 °C-annealed sample in the low temperature regime (100~250 °C). This result implies that the higher catalytic activity of 750 °C-annealed sample than that of 450 °C-annealed one at low temperature (~100 °C) was related to the fact that the reduction of 750 °C-annealed sample was more facile than 450 °C-annealed one. However, the exact origins of enhanced catalytic activity of 750 °C-annealed Fe_2_O_3_/Al_2_O_3_ are not clearly understood at this point, e.g., whether the difference in catalytic activity of 450 and 750 °C-annealed sample were determined by the number of surface Fe(III) states or Fe ion in lower oxidation states. This issue warrants further investigation using *in-situ* XPS analysis system equipped with a stage for temperature programmed reduction and oxidation experiments.

It can be suggested that the Fe_2_O_3_ showed a rather flat 2D structure after annealing at 450 °C and a more 3D structure formed as the annealing temperature increased from 450 to 750 °C (Supplementary Fig. 6). Due to higher electronegativity of Fe (1.8) than that of Al (1.5), charge transfer from Al to Fe can take place, which can be indicated by the positive shift of the Al 2p core-level peak after annealing at 450 °C (Fig. 5a). When the 3D growth of Fe_2_O_3_ nanoparticles took place as the annealing temperature increased from 450 to 750 °C, the metal-support charge transfer can become less pronounced, shifting the binding energy of Al 2p XPS peak to its original positions subjecting to no additional charges due to the metal-support interaction (Supplementary Fig. 6). Due to the Fe_2_O_3_ particle size change as well as structural alteration upon annealing at different temperature, information about metal-to-support charge transfer is not clearly extracted from the Fe 2p spectra. At the same time, decrease of the metal-support charge transfers with increasing annealing temperature from 450 to 750 °C led to the formation of more pronounced features for the surface Fe(III) species of bulk Fe_2_O_3_. It is worth to note that the relative Fe 2p peak intensity to the respective Al 2p peak decreased with increasing annealing temperature (from 450 to 750 °C). (Supplementary Table 2). It indicated that Fe_2_O_3_ nanoparticles underwent thermal aggregation at higher temperature resulting in the decrease of surface to volume ratio, which is also in line with the TEM images (Fig. 2).

**Figure 7:**
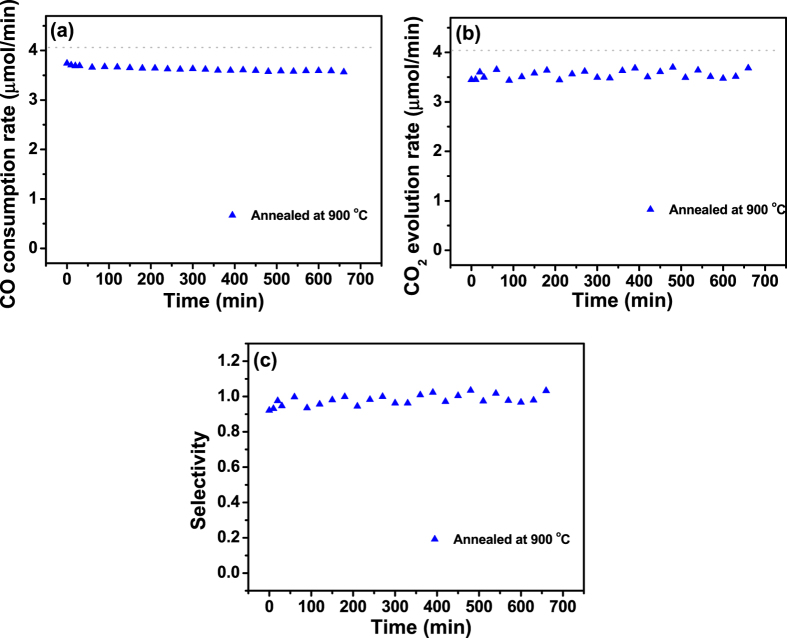
(**a**) CO consumption rate, (**b**) CO_2_ evolution rate, and (**c**) CO_2_ selectivity of Fe_2_O_3_/Al_2_O_3_ annealed at 900 °C. Dashed lines indicate the respective values corresponding to 100% conversion of CO to CO_2_.

In order to check the thermal stability of the Fe_2_O_3_/Al_2_O_3_ catalyst at higher temperature (>750 °C), its catalytic activity was evaluated after the annealing at 900 °C for 2 h (Fig. 7). The activity of Fe_2_O_3_/Al_2_O_3_ annealed at 900 °C was slightly lower than that of the Fe_2_O_3_/Al_2_O_3_ catalyst annealed at 750 °C. However, it still showed higher activity compared to the Fe_2_O_3_/Al_2_O_3_ annealed at 600 °C. This emphasizes that, unlike NiO/Al_2_O_3_ or NiO/SiO_2_ catalysts (which showed a significant reduction in their catalytic activity when the annealing temperature exceeded 450 °C), Fe_2_O_3_/Al_2_O_3_ can maintain its activity up to 900 °C^34^,^35^.

**Figure 8:**
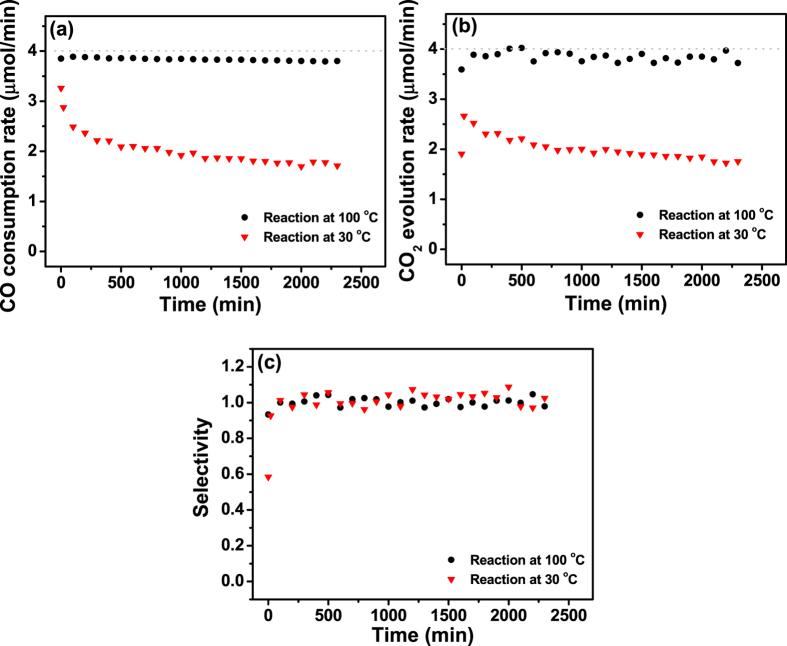
(**a**) CO consumption rate, (**b**) CO_2_ evolution rate, and (**c**) CO_2_ selectivity of Fe_2_O_3_/Al_2_O_3_ annealed at 750 °C for CO oxidation at 30 and 100 °C. Dashed lines indicate the respective values corresponding to 100% conversion of CO to CO_2_.

The long-term stability of the catalytic activity of Fe_2_O_3_/Al_2_O_3_ annealed at 750 °C was also tested at 30 and 100 °C for 40 h (Fig. 8). At 100 °C, the Fe_2_O_3_/Al_2_O_3_ catalyst annealed at 750 °C showed no significant deactivation, and ~95% of the activity was maintained for 40 h. Further on, it exhibited catalytic activity towards CO oxidation at 30 °C; ~60% of the CO consumption and CO_2_ evolution rate were observed at the early stage of the reaction. A significant drop of the catalytic activity was only observed at the initial stage of the reaction, whereas the activity was rather stable after ~250 min. It is worth noting that the catalytic activity of Fe_2_O_3_/Al_2_O_3_ at 30 °C is comparable with that of the NiO/Al_2_O_3_ catalyst prepared by ALD^34^,^35^.

When the surface of catalysts exposed to higher number of reactants per a unit time (higher space velocity of reactants) deactivation of catalytic activity can be pronounced at the same temperature where the catalyst showed no deactivation at lower space velocity. In order to address this issue, catalytic activity of Fe_2_O_3_/Al_2_O_3_ was evaluated with three different flow rate of reagents gas (10, 50, and 100 ml/min of dry air flow containing 1% CO) (Supplementary Fig. 7). As the flow rate of reagents increased, initial CO conversion efficiency of the sample gradually decreased from 82% (50 ml/min) to 63% (100 ml/min). However most importantly, the catalytic activity of the sample underwent only the minor deactivation even at the highest flow of reagents (100 ml/min); CO conversion efficiency only decreased by about 15% from its initial value after 600 min at 100 ml/min of reagents gas flow.

We compared the catalytic activity of Fe_2_O_3_/Al_2_O_3_ to NiO catalyst deposited via atomic layer deposition on the same mesoporous Al_2_O_3_^52^ (Supplementary Fig. 8). Here, the experimental conditions for the CO oxidation experiment (the amount of catalysts, gas composition, and flow rate) excepting for the reaction temperature were the same for both catalysts. Fe_2_O_3_/Al_2_O_3_ showed a higher catalytic activity and resistance toward deactivation even at a lower reaction temperature. The turn-over-frequency (TOF) of each catalyst for 680 min was estimated, assuming that both the NiO and Fe_2_O_3_ particles on mesoporous Al_2_O_3_ had a hemispherical structure. The calculation procedure of active sites of catalysts can be found in supplementary information. The TOF of NiO/Al_2_O_3_ at 150 °C was 1.0 × 10^−4^ s^−1^, while that of Fe_2_O_3_/Al_2_O_3_ at 100 °C was 7.4 × 10^−5^ s^−1^. It is important to note that the amount of Ni loading of ALD-prepared NiO/Al_2_O_3_ was only ~0.4 wt% due to the small NiO shell depth ~15 μm, which is one order of magnitude lower than the case of Fe_2_O_3_/Al_2_O_3_ with Fe loading of ~5 wt%^42^. However, it can be determined that the orders of magnitude of the TOF values of NiO/Al_2_O_3_ and Fe_2_O_3_/Al_2_O_3_ are still comparable. We’d also like to emphasize that NiO catalysts showed a gradual decrease in performance with increasing reaction time (even at 150 °C), whereas almost no deactivation of the Fe_2_O_3_/Al_2_O_3_ catalyst was found at 100 °C.

For comparison, the catalytic activity of a mechanically ground Fe_2_O_3_/Al_2_O_3_ towards CO oxidation was compared with those of two commercially available catalysts (Pt/AC, and γ-Fe_2_O_3_) in the temperature range of 300~50 °C (Supplementary Fig. 9). The CO conversion (%) of Pt/AC was slightly higher than Fe_2_O_3_/Al_2_O_3_ at reaction temperature higher than 200 °C. However, the catalytic activity of Pt/AC decreased drastically as the temperature decreased below 200 °C, and Fe_2_O_3_/Al_2_O_3_ showed higher catalytic activity at lower temperature range (<~180 °C) than Pt/AC catalyst. The CO conversion of Pt/AC was about 10% at 180 °C and the conversion of CO to CO_2_ was barely observed below 150 °C, while Fe_2_O_3_/Al_2_O_3_ still exhibited 50% of CO conversion at 150 °C. Over the entire region of the reaction temperature (50~300 °C), Fe_2_O_3_/Al_2_O_3_ catalyst showed higher activity than γ-Fe_2_O_3_ with same specific area of Fe_2_O_3_ at the same temperature.

In conclusion, we fabricated Fe_2_O_3_ nanostructures on mesoporous Al_2_O_3_ using the TR-CVD method under ambient conditions. With this new strategy to incorporate metal oxide nanoparticles into mesoporous media, Fe_2_O_3_ could even be deposited at the core region of Al_2_O_3_ (with a diameter of 1 mm), while maintaining the mesoporous structure of the substrate. Fe_2_O_3_ deposited on the surface of Al_2_O_3_ maintained its lateral size of ~1 nm after annealing at 750 °C in air. The catalytic activity for CO oxidation of Fe_2_O_3_/Al_2_O_3_ at 100 °C increased as the annealing temperature increased from 450 to 750 °C. The Fe_2_O_3_ catalyst annealed at 750 °C, which was the most active catalyst, showed high catalytic activity for CO oxidation even at 30 °C. The XPS data suggests that the surface Fe(III) species of bulk Fe_2_O_3_ could be the active species for CO oxidation. In addition, the Fe_2_O_3_/Al_2_O_3_ annealed at 750 °C maintained its catalytic activity for 40 h without significant deactivation at 100 °C. Our Fe_2_O_3_/Al_2_O_3_ catalysts also sustained their catalytic activity even after annealing at 900 °C. We suggest that TR-CVD is a simple and efficient way to incorporate metal oxide nanoparticles into mesoporous substrates. Supported Fe_2_O_3_ nanostructures prepared by TR-CVD, with sequential annealing treatments, are promising catalysts for catalytic oxidation reactions; these catalysts operate at room temperature, do not undergo deactivation at relatively low temperatures (~100 °C), and sustain their high catalytic activity even after very severe thermal treatment (~900 °C).

## Methods

### Sample preparation

The Fe_2_O_3_/Al_2_O_3_ catalysts were prepared by deposition of Fe_2_O_3_ on mesoporous Al_2_O_3_ (bead size: 1 mm, mean pore size: 11.6 nm, Sasol) using TR-CVD. For TR-CVD of Fe_2_O_3_, bis(cyclopentadienyl)iron (Fe(Cp)_2_, Aldrich) powder was used as a metal precursor and the oxygen (and water vapor) that is naturally present in air was used as the oxidizing agent. 2.5 g of Fe(Cp)_2_ was loaded in a quartz boat located on the bottom of the chamber, and 10 g of Al_2_O_3_ (on a metal mesh) was placed above the Fe(Cp)_2_. First, in order to vaporize Fe(Cp)_2_, the temperature of the chamber was increased to 60 °C; this temperature was maintained for 2 h. After 2 h, the temperature of the chamber was further elevated to 200 °C and maintained for about 12 h. More detailed descriptions on the TR-CVD system can be found in Supplementary Information (Supplementary Fig. 10).

### Sample characterization

After deposition of Fe_2_O_3_ using TR-CVD, the elemental distribution of the cross-sectional plane of Fe_2_O_3_/Al_2_O_3_ was analyzed by SEM (JEOL, JSM-7100F) equipped with EDS. Fe_2_O_3_/Al_2_O_3_ samples were mechanically fractured to analyze their internal structure. The elemental contents of Fe_2_O_3_/Al_2_O_3_ were analyzed by ICP-OES (Varian). After annealing the Fe_2_O_3_/Al_2_O_3_ at four different temperatures (450, 600, 750, and 900 °C), N_2_ adsorption/desorption isotherms were obtained for four samples using a gas sorption analyzer (3Flex, Micromerities) and the average pore diameter and surface area of each Fe_2_O_3_/Al_2_O_3_ sample were estimated based on BJH and BET method. The detailed structures of the Fe_2_O_3_ particles on Al_2_O_3_ after the annealing at 450 and 750 °C under dry air condition were investigated using HAADF STEM (JEOL, JEM ARM 200F). Further on, the surface chemical states of the Fe_2_O_3_/Al_2_O_3_ samples annealed at 450, 600, and 750 °C under the dry air flow were analyzed by XPS. The samples were annealed in a reactor inside the glove box filled with Ar, then transferred to the ultra-high vacuum (UHV) system using a magnetic transfer system (filled with Ar) without exposing the samples to the atmosphere. It prevents the possible contamination between the annealing process and XPS analysis. XPS analysis was carried out in the UHV chamber (base pressure of 3 × 10^−10^ Torr) using Mg Kα X-ray line (1253.6 eV) and a concentric hemispherical analyzer (CHA, PHOIBOS-HAS 3500, SPECS) at room temperature.

### Catalytic activity test

The catalytic activity of Fe_2_O_3_/Al_2_O_3_ for CO oxidation was studied in a continuous flow quartz reactor (internal diameter of 21 mm, length of 300 mm) located in the glove box. 2.0 g of catalyst was loaded into a quartz boat (internal volume of 70 × 20 × 8 mm^3^), and the boat was placed in the middle of the quartz reactor. Prior to each CO oxidation experiment, Fe_2_O_3_/Al_2_O_3_ samples were annealed at four different temperatures (450, 600, 750, and 900 °C) and annealing conditions at each temperature were specified in Supplementary Information. Dry air (21% of O_2_, balanced with N_2_) containing 1% of CO was fed into the reactor at a total flow of 10 ml/min during the reaction. Only the data points after 50 min are displayed here since it took 50 min to saturate the CO with a gas flow rate of 10 ml/min. The composition of gas passed the catalyst was analyzed by on-line gas chromatography (GC, Hewlett Packard, HP 6890) equipped with a capillary column (Agilent Technologies, HP-PLOT/Q, 30 m × 0.53 μm), a methanizer and a flame ionization detector (FID). The catalytic activities for CO oxidation of the Fe_2_O_3_/Al_2_O_3_ catalysts annealed at 450, 600, 750, and 900 °C were measured at 100 °C for 12 h. The long-term catalytic activity of the Fe_2_O_3_/Al_2_O_3_ annealed at 750 °C exhibiting the highest activity at 100 °C for 12 h was also studied at 30 and 100 °C for 40 h. Also, the catalytic activity at higher space velocity (50 and 100 ml/min) of 1% CO/air gas were tested. The CO consumption and CO_2_ evolution rates were calculated by using the same method as our previous work^34^,^35^.

Two commercially available catalysts, platinum supported by activated carbon with 5% of Pt loading (a specific surface area 500~1200 m^2^/g) and Fe_2_O_3_ nanopowder (γ-Fe_2_O_3_, <50 nm), were bought from Sigma Aldrich, and their catalytic activities towards CO oxidation were evaluated over the reaction temperature range of 50~300 °C under same experimental conditions used for the case Fe_2_O_3_/Al_2_O_3_ catalyst. Their catalytic activities were compared with that of mechanically ground Fe_2_O_3_/Al_2_O_3_ sample and the amount of each catalyst was carefully chosen to adjust the surface area of active metal nanoparticles at same level. The amount of each catalyst, annealing conditions, and conditions of CO oxidation activity test were specified in Supplementary Information.

### H_2_-temperature programmed reduction (H_2_-TPR)

H_2_-TPR experiments were also performed using a continuous flow quartz reactor which was used for catalytic activity test. 2.0 g of catalysts were annealed in the reactor at two different temperatures (450 and 750 °C) for 2 h. And then the reactor was cooled down to 30 °C, the H_2_ and N_2_ gases were fed into the reactor using mass flow controllers at constant flow rates of 2 ml/min (H_2_) and 18 ml/min (N_2_) during the TPR experiment. After the H_2_ level reached to its saturation level, we began to increase the temperature of the reactor from 30 to 450 °C with a constant rate of 1 °C/min, and H_2_ uptake was monitored by on-line gas chromatography (GC, Hewlett Packard, HP 6890) equipped with a thermal conductivity detector (TCD).

## Additional Information

**How to cite this article**: Kim, I. H. *et al*. Low Temperature CO oxidation over Iron Oxide Nanoparticles Decorating Internal Structures of a Mesoporous Alumina. *Sci. Rep.* **7**, 40497; doi: 10.1038/srep40497 (2017).

**Publisher's note:** Springer Nature remains neutral with regard to jurisdictional claims in published maps and institutional affiliations.

## Supplementary Material

Supplementary Information
