# Supplementary material for: Low Temperature CO oxidation over Iron Oxide Nanoparticles Decorating Internal Structures of a Mesoporous Alumina
Source: Sci Rep. 2017 Jan 16;7:40497. doi: 10.1038/srep40497 (PMC5238452; doi:10.1038/srep40497)
Supplement: Supplementary Information [file srep40497-s1.pdf]

Supplementary Information

Low temperature CO oxidation over iron oxide nanoparticles decorating internal structures of a mesoporous alumina

Il Hee Kim[1], Hyun Ook Seo[1*], Eun Ji Park[1], Sang Wook Han[1], and Young Dok Kim[12*]

[1]Department of Chemistry, Sungkyunkwan University, Suwon, 440-746, Republic of Korea

[2]Research Center for Nanocatalysts, Korea Research Institute of Chemical Technology (KRICT), Daejeon 305-600, Republic of Korea

Corresponding author

[*]E-mail: ydkim91@skku.edu (Y. D. Kim),
seoho83@skku.edu (H. O. Seo).

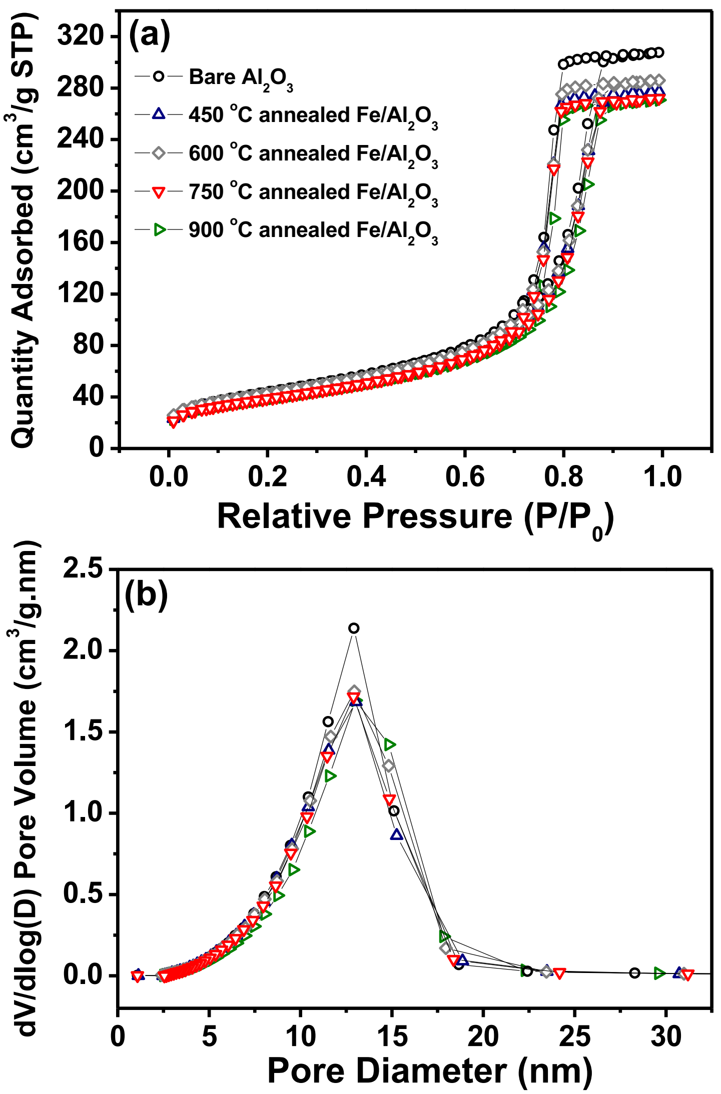

Supplementary Figure 1.

(a) N_2 adsorption/desorption isotherm and (b) pore diameter distribution of bare Al_2O_3, Fe/Al_2O_3 annealed at 450, 600, 750, and 900 ^oC. After Fe_2O_3 deposition and post-annealing up to 900 ^oC, the porous structure of the samples did not showed significant changes.

	Surface area (m^2/g)	Pore diameter (nm)
Bare Al_2O_3	158.0	12.1
Fe_2O_3/Al_2O_3 annealed at 450 ^oC	146.4	11.7
Fe_2O_3/Al_2O_3 annealed at 600 ^oC	151.8	11.6
Fe_2O_3/Al_2O_3 annealed at 750 ^oC	139.2	12.1
Fe_2O_3/Al_2O_3 annealed at 900 ^oC	137.8	12.1

Supplementary Table 1.
Surface area and average pore diameter of bare Al_2O_3, Fe/Al_2O_3 annealed at 450, 600, 750, and 900 ^oC.

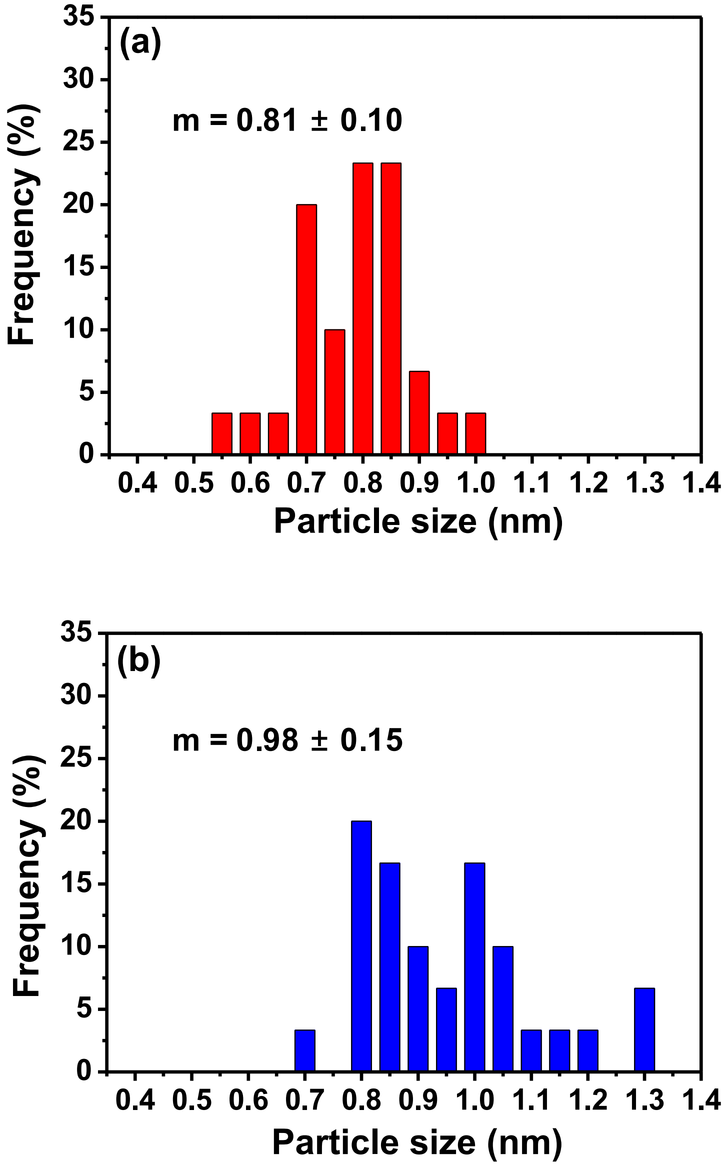

Supplementary Figure 2.
Size distribution of Fe_2O_3 nanoparticles on Fe_2O_3/Al_2O_3 catalysts annealed at (a) 450 and (b) 750 ^oC.

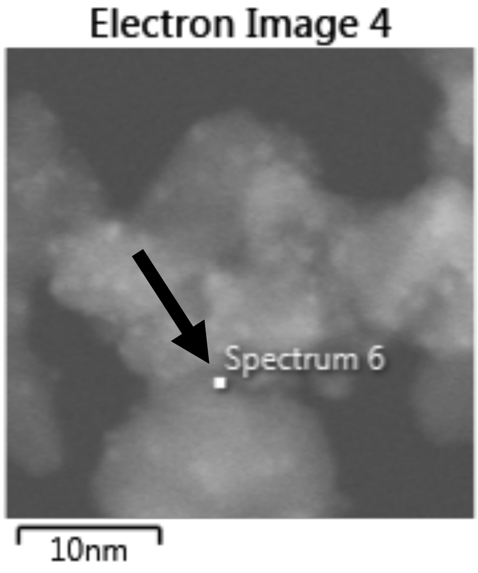

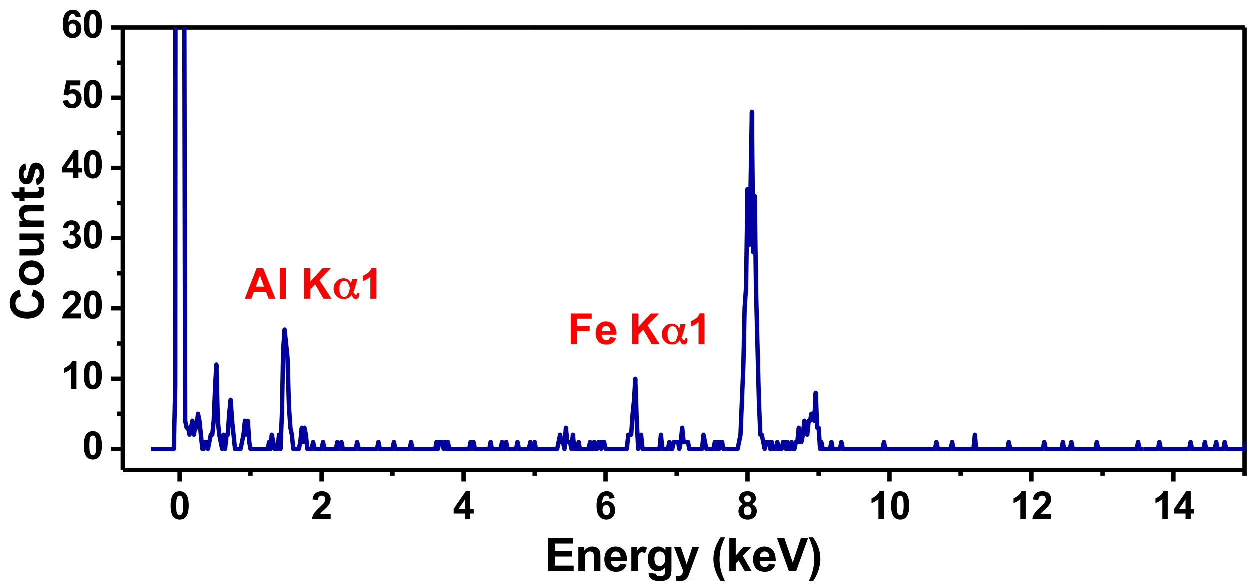

Element	Line Type	k factor	Absorption Correction	Wt%	Wt% Sigma	Atomic %
**Al**	K series	1.06300	1.00	55.43	6.47	72.02
**Fe**	K series	1.07637	1.00	44.57	6.47	27.98
**Total:**				100.00		100.00

Supplementary Figure 3.
The results of EDS (Energy dispersive spectroscopy) mapping of 750 ^{o}C-annealed Fe_2O_3/Al_2O_3 catalyst. The area taken for EDS analysis is marked with white square dot guided by black arrow.

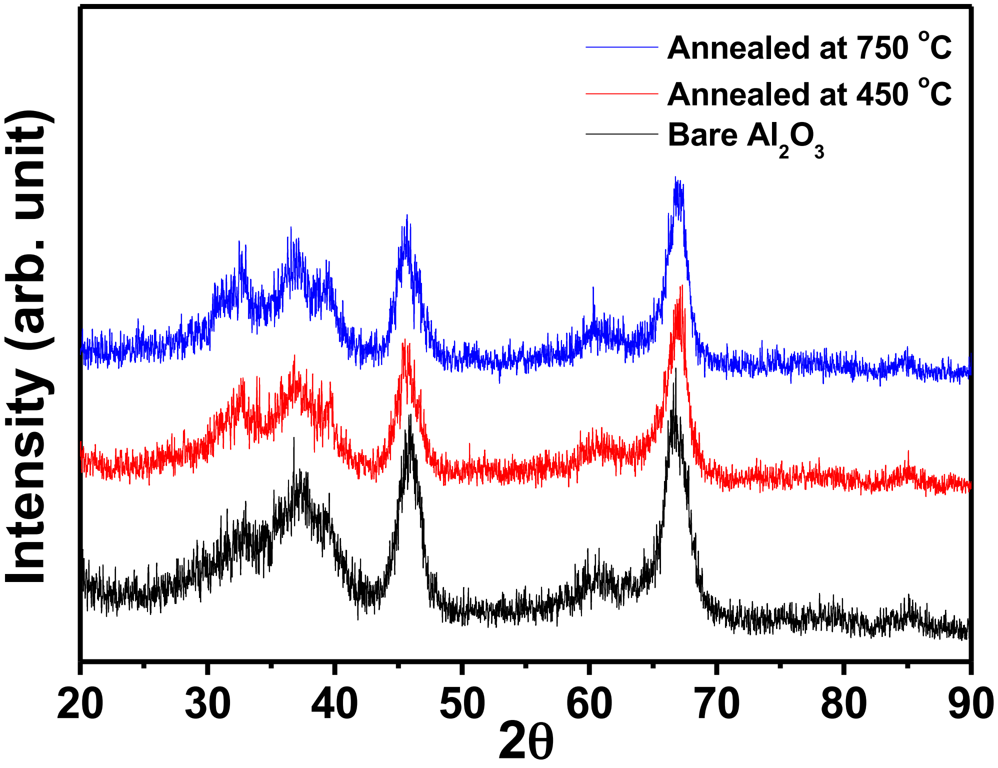

Supplementary Figure 4.
XRD pattern of bare Al_2O_3, Fe-deposited Al_2O_3 annealed at 450 and 750 ^oC.

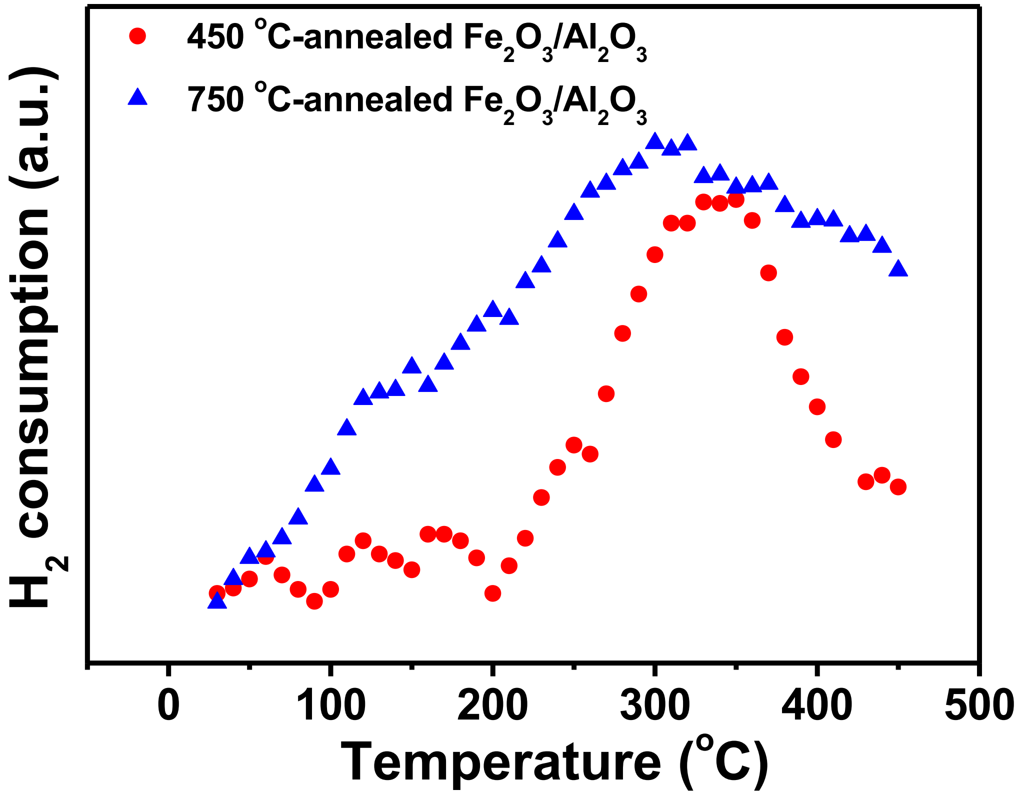

Supplementary Figure 5.
The amount of H_2 uptake of two Fe_2O_3/Al_2O_3 catalyst annealed at 450 and 750 ^oC during the H_2-TPR experiments.

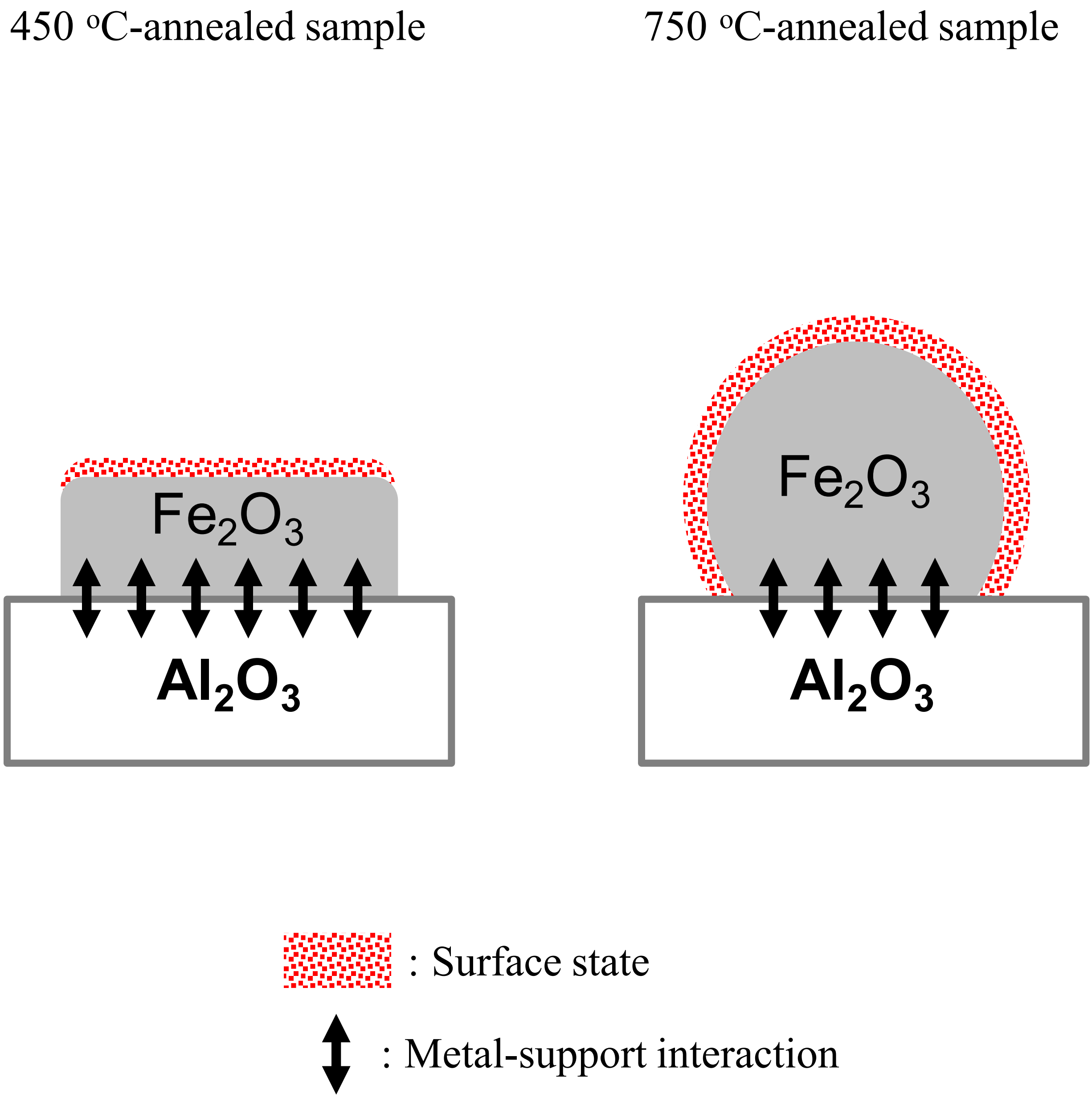

Supplementary Figure 6.
The schematic descriptions of different degree of metal-support charge transfer and different portion of surface Fe (III) states to bulk Fe (III) states between two Fe_2O_3/Al_2O_3 catalyst annealed at 450 and 750 °C, originating from the 3D growth of Fe_2O_3 nanoparticles with increasing annealing temperature from 450 to 750 °C.

Pre-annealing temperature	Fe/Al
450 ^{o}C	0.53
600 ^{o}C	0.44
750 ^{o}C	0.37

Supplementary Table 2.
Fe/Al ratio on the surface as a function of post-annealing temperature determined by XPS

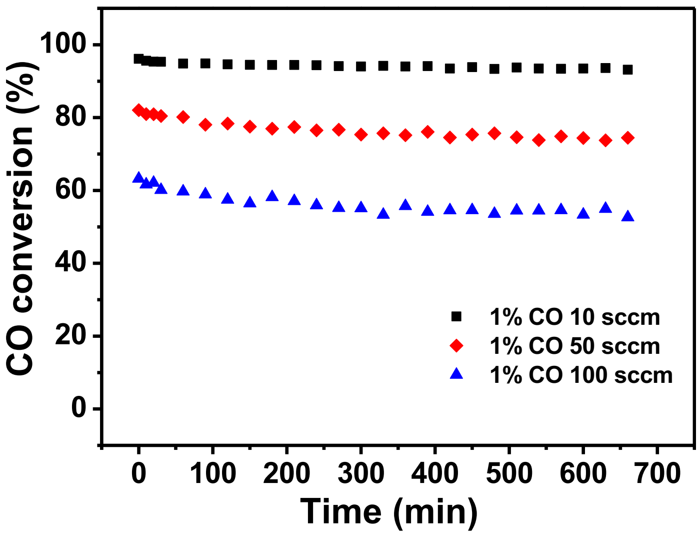

Supplementary Figure 7.
CO conversion of Fe_2O_3/Al_2O_3 with different space velocity of 1 % CO

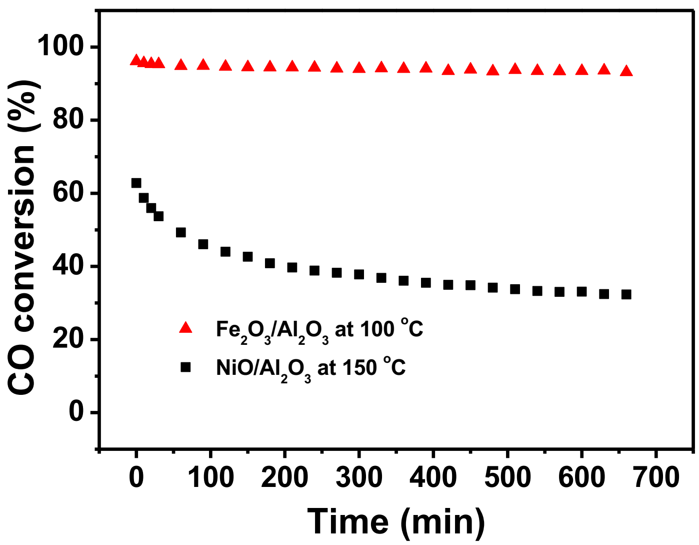

Supplementary Figure 8.

Catalytic activity of Fe_2O_3/Al_2O_3 (TR-CVD prepared) at 100 ^oC and NiO/Al_2O_3 (ALD-prepared) at 150 ^oC. The flow rate of dry air containing 1 % CO was 10 ml/min for both experiments.

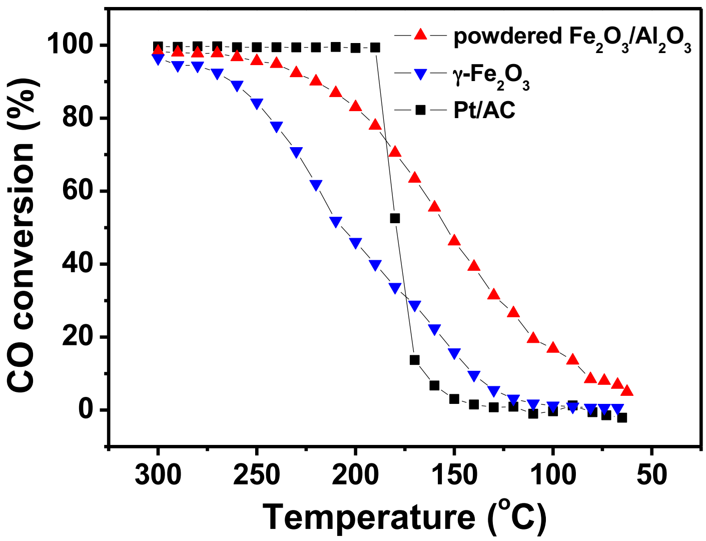

Supplementary Figure 9.
Catalytic activity of powdered Fe_2O_3/Al_2O_3, γ-Fe_2O_3, and Pt/AC as a function of reaction temperature. The amount of samples were adjusted in order to get equivalent specific area of active metal nanoparticles.

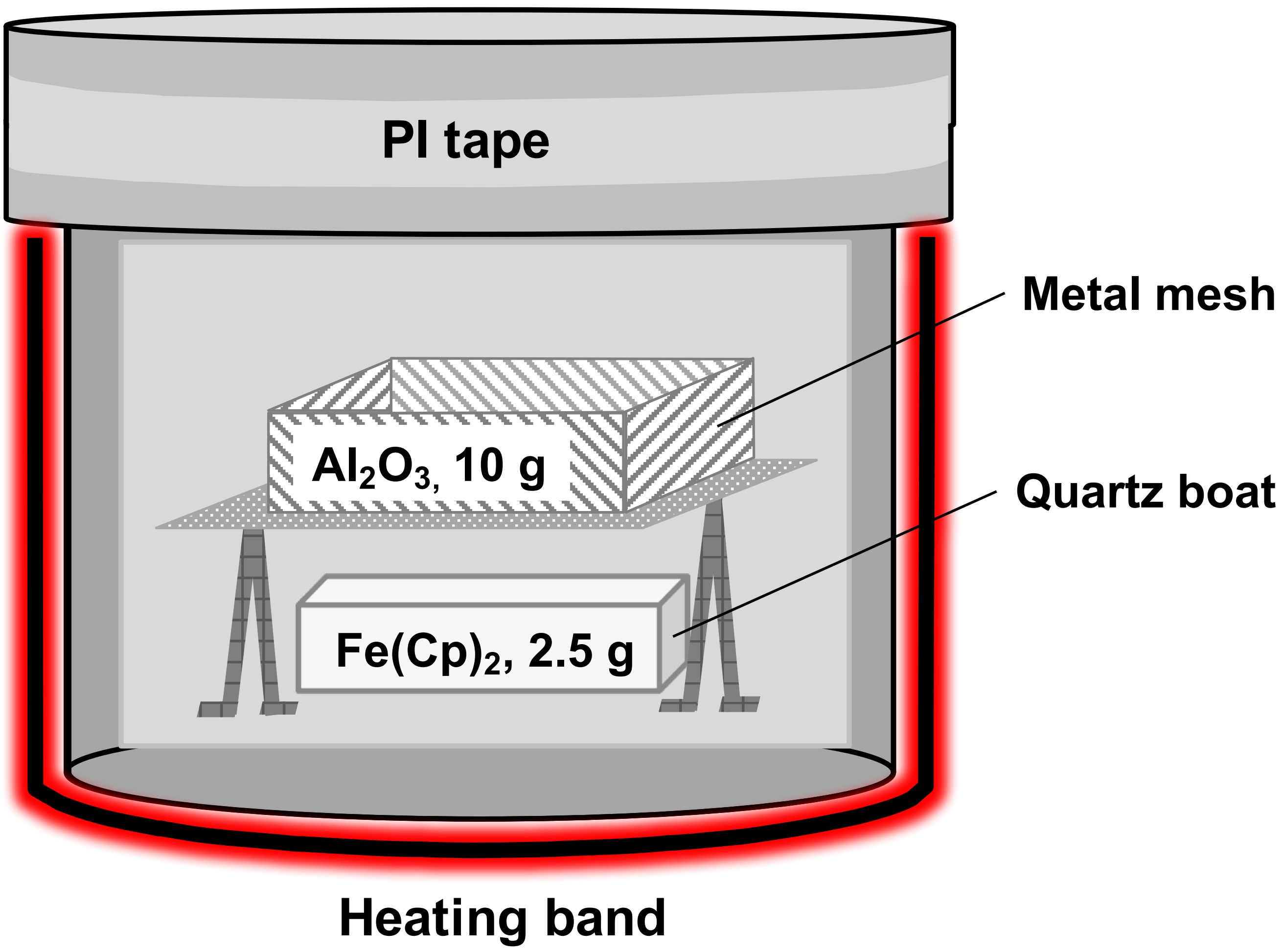

Supplementary Figure 10.
A Schematic diagram of the experimental set-up of TR-CVD for sample preparation. The gap between the lid and and the body of the chamber was sealed with polyimide (PI) film from the outside. The system was equipped with a thermocouple and heating jacket, wrapping the outside the chamber, for temperature control.

Conditions of the post-annealing of Fe_2O_3/Al_2O_3 samples.

Prior to the CO oxidation experiments, Fe_2O_3/Al_2O_3 samples were annealed at four different temperatures (450, 600, 750 and 900 ^oC). Annealing process at 450, 600, and 750 ^oC were conducted for 2 hrs under dry air conditions (total flow of 30 ml/min) in the reactor. On the other hands, the annealing process at 900 ^oC were done under ambient conditions (outside of the glove box) for 2 hrs and then transferred into the reactor inside the glove box. Then, the 900 ^oC-annealed sample was thermally treated again at 750 ^oC before the CO oxidation experiment in the glove box.

Conditions of CO activity tests of two commercially available catalyst (Pt/Ac, Fe_2O_3 nanopowder).

0.45 g of Pt/AC, 0.041 g of γ-Fe_2O_3, and 0.11 g of ground Fe_2O_3/Al_2O_3 were used for catalytic activity test. The powder catalysts were sandwiched using quartz wool and located in the middle of the quartz tube. Prior to CO oxidation experiment, the Pt/AC sample was annealed at 450 ^oC for 2 h under dry air condition (total flow of 30 ml/min), while γ-Fe_2O_3, and 0.11 g of ground Fe_2O_3/Al_2O_3 samples were annealed at 750 ^oC under same conditions (2 hr, 30 ml/min of air flow). After the annealing processes, the reactor was cooled down to 300 ^oC, then the 1 % CO gas was fed into the reactor with flow rate of 10 ml/min. The temperature of the reactor was kept at 300 ^oC for 2 h, then cooled down to 50 ^oC with a constant cooling rate of 1 ^oC/min, and the CO oxidation activity was measured during the cooling process.

Calculation of active sites on the surface of Fe_2O_3/Al_2O_3 catalyst (2.0 g) for TOF estimation.

We assumed that the reaction was in thermodynamic controlled regime and TOF values were calculated using the number of consumed CO molecules (mol) averaged over the reaction time. For the calculation of the number of active sites of TR-CVD prepared Fe_2O_3/Al_2O_3 catalyst, we assumed the hemispherical shape of Fe_2O_3 nanoparticles on Al_2O_3 substrate and 2 active sites per (200) plane of Fe_2O_3. In the below, we summarized the process of the calculation of active sites number together with important parameters used for the calculation.

Value	Estimated number	Determined by
The weight percent of Fe atoms in Fe_2O_3/Al_2O_3.	5.39 wt%	ICP-Mass
The weight of Fe atoms in 2.0 g of Fe_2O_3/Al_2O_3.	0.108 g	the total weight of Fe_2O_3/Al_2O_3 catalyst multiplied by the weight percent of Fe atoms in Fe_2O_3/Al_2O_3.
The radius of Fe_2O_3 nanoparticles	0.5 nm	TEM analysis
Density of Fe_2O_3	5.242 g/cm^3	
The unit cell area of Fe_2O_3 (200) plane	0.706 nm^2	
The volume of a Fe_2O_3 particle	2.618×10^{-22} cm^3	the volume of a spherical particle of Fe_2O_3 multiplied by 1/2
The mass of a Fe_2O_3 particle	1.372×10^{-21} g	the volume of a Fe_2O_3 particle multiplied by density of Fe_2O_3
The number of Fe_2O_3 particles in 2.0 g of Fe_2O_3/Al_2O_3	1.123×10^{20}	the weight of Fe atoms in 2.0 g of Fe_2O_3/Al_2O_3 divided by the mass of a Fe_2O_3 particle
The surface area of a Fe_2O_3 particle	1.571 nm^2	the surface area of a spherical particle of Fe_2O_3 multiplied by 1/2
The total surface area of Fe_2O_3 particles in 2.0 g of Fe_2O_3/Al_2O_3	1.7641×10^{20} nm^2	the surface area of a Fe_2O_3 particle multiplied by the number of Fe_2O_3 particles in 2.0 g of Fe_2O_3/Al_2O_3
The number of unit cell of Fe_2O_3 (200) plane	2.5×10^{20}	the total surface area of Fe_2O_3 particles in 2.0 g of Fe_2O_3/Al_2O_3 divided by the unit cell area of a Fe_2O_3 (200) plane
The number of active sites on the surface of Fe_2O_3/Al_2O_3 (2.0 g)	5.0×10^{20}	the number of unit cell of Fe_2O_3 (200) plane multiplied by the number of active sites per a unit cell of Fe_2O_3 (200) plane
